# Online ACT intervention for fibromyalgia: An exploratory study of feasibility and preliminary effectiveness with smartphone-delivered experiential sampling assessment

**DOI:** 10.1016/j.invent.2022.100561

**Published:** 2022-07-08

**Authors:** Pablo de la Coba, Miguel Rodríguez-Valverde, Mónica Hernández-López

**Affiliations:** Department of Psychology, University of Jaén, Spain

**Keywords:** Acceptance and commitment therapy, Chronic pain, Experiential sampling, Fibromyalgia, Processes of change, Psychological flexibility

## Abstract

**Introduction:**

Acceptance and commitment therapy (ACT) is an effective treatment for chronic pain conditions. ACT seeks to produce clinical change by enhancing Psychological Flexibility (PF). This exploratory (feasibility and preliminary effectiveness) study presents a pilot application of an online ACT group intervention for fibromyalgia (FM) with an extensive Experiential Sampling (ES) assessment of outcome and process variables via smartphone.

**Method:**

5-weekly ACT online group sessions were applied to 9 female FM patients. Questionnaire-based assessments of several clinical outcomes and PF processes were conducted pre- and post-intervention, and at 6-month follow-up. Extensive (6 weeks pre- and 6 weeks post-intervention) smartphone-delivered ES was implemented to gather process and outcome data in the patients' usual contexts. Clinically significant change was evaluated both at the group level and individually.

**Results:**

This treatment format appears to be feasible and acceptable to participants, with good adoption and completion rates (75 %) and excellent rates of treatment completion and clinical adherence (100 %). Participants showed significant reductions in affective pain, distress and biopsychosocial impact of FM both post-intervention and at 6-month follow-up (as measured with questionnaires), as well as significant improvements in satisfaction with actions and emotional discomfort (as measured through ES). Multilevel regression analyses indicated that PF-related processes assessed through ES had a significant impact on clinical outcomes and predicted the impact of FM at the 6-month follow-up.

**Conclusions:**

A brief online group ACT intervention for FM was both feasible and acceptable to participants. Also, there was preliminary evidence of effectiveness in enhancing pain-related PF and producing clinical benefits in FM.

## Highlights

• An online ACT intervention shows a great preliminary effectiveness in fibromyalgia.
• Experiential Sampling enhances the understanding of the processes of clinical change.
• Smartphones makes possible the collection of data in the patients' usual contexts.
• Psychological Flexibility assessed in usual contexts is key for the clinical change.

## 1 Introduction

Fibromyalgia (FM) is a chronic pain syndrome characterized by physical symptoms like fatigue, stiffness or insomnia, and psychological alterations such as anxiety or depression (de la Coba et al., 2020; Wolfe et al., 1990, Wolfe et al., 2010) that limits the life of people suffering from it. FM is currently considered an irresolvable primary chronic pain condition (Treede et al., 2019). Psychological intervention appears as a key component of chronic pain management, with cognitive-behavioral therapy (CBT) and acceptance and commitment therapy (ACT) as two of the most recommended and evidence-supported approaches (Bernardy et al., 2010; Hann and McCracken, 2014; Williams et al., 2020).

The use of internet-delivered psychological interventions has grown exponentially in the last years (Andersson, 2016; Zale et al., 2021), with a substantial acceleration due to the COVID-19 pandemic (Wind et al., 2020). Social and mobility restrictions adopted in response to the pandemic have had a large negative impact on population mental health (Clarfield and Jotkowitz, 2020), as well as hindered access to face-to-face psychological attention services. This situation has evidenced the need for flexible treatment-delivery alternatives that reach larger numbers of people in need of assistance (Philippi et al., 2021). Precisely, some of the main advantages of online interventions are their accessibility and acceptability for individuals and groups that would not otherwise engage in face-to-face treatment (e.g. cases of social isolation, stigmatization, fear of physical contact, difficulties with schedule or travelling costs) (see: Andersson and Titov, 2014; Baumeister et al., 2017). Online psychological interventions for chronic pain have amply demonstrated to be effective (Buhrman et al., 2016), with similar effects of face-to-face and online formats (Carlbring et al., 2018; Herbert et al., 2017). CBT and ACT appear as the internet-delivered therapies with greater evidence (Eccleston et al., 2014; van de Graaf et al., 2021), achieving significant improvements in quality of life, moderate reductions of distress, and modest reductions of pain-related symptoms in chronic conditions, including FM (Bernardy et al., 2019; Trindade et al., 2021; White et al., 2020). Although online interventions have demonstrated to be effective even in one-session and in a self-help format, their effectiveness appears to increase with a minimum of sessions and in a guided format (Lin et al., 2015; Vowles et al., 2020).

The effectiveness of ACT for chronic pain conditions is well established (Galvez-Sánchez et al., 2021; Vowles et al., 2020). In addition, ACT has a clear focus on the processes that underlie clinical improvement (Åkerblom et al., 2021; McCracken and Vowles, 2014; Scott et al., 2016; Vowles et al., 2009; Yu et al., 2017). For FM, ACT has proved to be effective both in online (Ljótsson et al., 2014; Simister et al., 2018) and face-to-face interventions (J. Luciano et al., 2014; Jensen et al., 2012; Wicksell et al., 2013). Specifically, ACT seeks to produce clinical change by enhancing Psychological Flexibility (PF) (Hayes et al., 2012; Vowles et al., 2014). PF in chronic pain patients could be defined as the ability to fully experience one's pain and any other private events that may arise while acting effectively in accordance with personal values. PF consists of six core sub-processes that can be pragmatically grouped into three dyadic processes or response styles (Hayes et al., 2011, Hayes et al., 2012). The Open response style (comprising the processes of acceptance and defusion) involves openness to experience and detachment from literality. The Aware/Centered response style (comprising self-as-context and present moment awareness) involves a focus on the present moment as a conscious person. The Active/Engaged response style (comprising values and committed action) involves behavioral activation towards a meaningful, valued life. Evidence shows that changes in PF processes underlie clinically relevant changes in chronic pain patients (Åkerblom et al., 2021; Costa and Pinto-Gouveia, 2011; Scott et al., 2016; Solé et al., 2016; Yu et al., 2017), in line with a process-based therapy approach (Hofmann and Hayes, 2019).

Different researchers have recently pointed out the limitations of analyzing processes of clinical change by exclusively relying on a nomothetic framework, with standardized measures of process and outcome variables collected in situations removed from the individual's natural context (Ciarrochi et al., 2021; Lavefjord et al., 2021). It has been suggested that researchers consider a more idiographic approach in order to identify underlying processes of clinical change, using high temporal density and contextualized measurement applied at the level of the person (Ciarrochi et al., 2021; Hofmann and Hayes, 2019). A combination of both approaches seems desirable in order to appreciate the complete picture, considering that results after an intervention can differ at the individual and the group level (e.g. Schmidt et al., 2015).

A suitable assessment strategy in this regard is Experiential Sampling (ES). ES is an evaluation method based on the collection of multiple reports about thoughts, feelings or manifest behaviors that occur on different situations during a determined time period in the natural environment of the person (Csikszentmihalyi and Larson, 2014). The widespread use of smartphones and other portable devices has rendered ES a particularly advantageous procedure in clinical studies (Bell et al., 2018; Nap-van der Vlist et al., 2021). ES has several advantages compared to the more traditional assessment based on standardized questionnaires: (a) greater sensitivity to clinical changes (Moore et al., 2016), (b) greater ecological validity, evaluating in the participant's natural context (Csikszentmihalyi and Larson, 2014) and collecting more representative information (Myin-Germeys et al., 2009), (c) lower measurement error and higher statistical power (Stone et al., 2007), (d) lower reactivity to evaluation (Myin-Germeys et al., 2018), and (e) less reliance on the individual's memory (Shiffman et al., 2008). Random temporal ES is usually the most advantageous, obtaining information at randomly variable time intervals (Myin-Germeys et al., 2018).

The clinical utility of ES for improving the assessment of clinical change and evolution has been demonstrated with patients with major depression or psychotic disorders (van Os et al., 2017), or with chronic pain conditions (Suso-Ribera et al., 2018). Specifically, its feasibility and usability through mobile devices seems promising in FM research and clinical practice (Castilla et al., 2012; Garcia-Palacios et al., 2014). Despite this, the extant evidence about the smartphone-based application of ES with chronic pain patients is still limited (Lavefjord et al., 2021). Specifically, regarding the application of ES in ACT studies, the findings from Villanueva et al. (2019) support the use of ES for a greater understanding of the mechanisms underlying ACT treatment success and failure in transdiagnostic patients (e.g. suffering from affective, anxiety, mood, or obsessive-compulsive disorders, amongst others). Additionally, ES has been used with ACT not only for assessment, but also for intervention, as a means of fostering awareness in the daily life of mental health inpatients (Batink et al., 2016). Finally, a recent application of ES in an ACT intervention for FM presented acceptable preliminary feasibility data (Gómez-Pérez et al., 2020). The authors of this study pointed out that, despite their promising findings, more research on single-case designs and patient-centered analyses was necessary (Gómez-Pérez et al., 2020).

The present study aimed to: [1] examine: [1A] the feasibility and [1B] preliminary effectiveness (both at a group level, and individually) of a group ACT intervention for FM patients delivered via online video meetings, with both questionnaire-based and contextualized (smartphone-based ES) assessments of clinical outcome indicators and PF-related processes; [2] determine what accounts for more variability in clinical outcomes as assessed via ES, whether (a) PF-related processes assessed in the participants' natural context (also via ES) or (b) clinical outcomes themselves as evaluated via standard questionnaires; and [3] examine whether the post-intervention PF-related processes in the participants' natural context can predict clinical outcomes at the 6-month follow-up.

## 2 Material and methods

### 2.1 Participants

All participants were recruited from a list of research-volunteer patients associated with a local FM patient-support association (the Fibromyalgia Association of Jaén, AFIXA). Participants were contacted by phone and recruited between October and November 2020. It is worth noting that the standing COVID-19 restrictions at the time precluded the possibility of face-to-face interaction for assessment or treatment with research purposes. Inclusion criteria were: being at least 18 years old, having FM diagnosis with at least a 5-year history of chronic pain, being willing to attend treatment and follow-up sessions and complete surveys, and having a broadband internet connection for participation in the online video meetings. Diagnosis was confirmed by review of medical records voluntarily provided by participants, by checking the fulfillment of the 1990 American College of Rheumatology criteria for FM (detection of pain to pressure in at least 11 of 18 tender points; see Wolfe et al., 1990). Exclusion criteria were: suffering any severe medical condition different to FM (e.g. cancer, stroke), suffering a major neuropsychiatric disorder (e.g. psychosis, dementia), presenting a substance use disorder. Sixteen potential FM patients (all female) were contacted by phone, 12 of which initially agreed to participate in the study. One of them refused to participate one week before the beginning of the study due to personal circumstances. Another two completed the pre-intervention assessment, but were excluded from analyses due to an impossibility to follow treatment in the same conditions as the rest of the group (they underwent the death of a first-degree relative, which interfered with session attendance). However, for ethical reasons they were allowed to stay in the group and received further individual psychological support. Therefore, the final sample consisted of 9 women suffering from FM with the following sociodemographic profile: 50.11 ± 4.96 years of age, married (100 %), mother (100 %), with secondary or higher education (89 %), currently employed (67 %) and assuming main responsibility for housekeeping (100 %) in their household. Regarding their chronic pain experience, participants presented a clinical history of 22.67 ± 7.38 years of pain, 9.33 ± 5.24 years since they were diagnosed with FM. Additionally, prolonged use (>2 years) of analgesics (89 %), antidepressants and/or anxiolytics (67 %), and previous experiences of psychological-support and/or physiotherapeutic treatments were reported (100 %), with little or no long-term self-reported clinical improvement as consequence of these.

### 2.2 Design

A pre-post design with a 6-month post-intervention follow-up was used to explore the feasibility and preliminary effects of an ACT group-online intervention in FM patients. In addition to the formal use of self-report questionnaires at pre- and post-intervention and follow-up, multiple measures were collected in the patients' natural contexts through Experiential Sampling (ES) for six weeks pre-and post-intervention. Besides, in order to promote compliance, the characteristics of FM patients were taken into account in designing the ES assessment (Van de Graaf et al., 2021). Instead of the typical ES configuration with multiple daily measurements, the current procedure involved multiple weekly measurements, which was aimed to prevent noncompliance due to fatigue. Finally, potential associations between post-intervention clinical outcomes and PF-related processes were also examined through selective analytical methodology.

### 2.3 Instruments and measures

#### 2.3.1 Standard questionnaires and scales

All standardized questionnaires were administered by telephone before and after the intervention, as well as in a 6-month follow-up.

### 2.4 Outcome variables

• McGill Pain Questionnaire (MPQ; Melzack, 1975), Spanish version (Lázaro et al., 1994). The MPQ has several scales to evaluate different dimensions of clinical pain. In order to obtain a general evaluative rating of clinical pain and assess its emotional component in a reliable and simple way, the present study used the evaluative (MPQ-E) and affective (MPQ-A) subscales. The MPQ-A consists of the following pain descriptors (multiple selection): annoying, worrying, agonizing, tiring, killing, nauseating, fearful, frightful, and terrifying (score range 0–9); while in the MPQ-E the patient has to select one of the following pain descriptors: weak, endurable, intense and unbearable (score range 1–4). For the complete instrument, Cronbach's α = 0.74, whilst for the MPQ-A subscale, α = 0.56 (Masedo and Esteve, 2000).
• Hospital Anxiety and Depression Scale (HADS; Zigmond and Snaith, 1983), Spanish version (Caro-Gabalda and Ibáñez, 1992). The HADS evaluates anxiety and depression in clinical populations with two subscales (HAS and HDS, respectively). It presents 14 items with 4 response options (scored 0 to 3). Higher scores indicate greater anxiety-depression symptoms. It has shown high reliability (Cronbach's α = 0.86) and validity (Quintana et al., 2003).
• Fibromyalgia Impact Questionnaire (FIQ; Burckhardt et al., 1991), Spanish validation (Rivera and González, 2004). It consists of 10 items that assess the severity of FM impact in the person's life. It is recommended for the evaluation of FM patients in clinical trials (Boomershine, 2012). Higher scores are indicative of greater impairment in individual functioning because of FM. Reliability and validity of the Spanish version are similar to the original version's, with a Cronbach's α = 0.82 and a test-retest reliability of 0.61–0.85 in intraclass correlations (Rivera and González, 2004).

### 2.5 Process variables

• Acceptance and Action Questionnaire - II (AAQ-II; Bond et al., 2011), Spanish version (Ruiz et al., 2013). The AAQ-II assesses the degree of experiential avoidance through questions related to unwillingness to experiencing emotions and the inability to behave according to personal values. It consists of 7 items rated on a Likert-type scale (1: never true – 7: always true). The Spanish version shows high reliability, Cronbach's α = 0.91 (Ruiz et al., 2016).
• Cognitive Fusion Questionnaire (CFQ, Gillanders et al., 2014), Spanish version (Ruiz et al., 2017). It assesses the degree of fusion of a person with their private events, as well as the ability to take distance from them. It consists of 7 items rated on a Likert-type scale (1: never – 7: always). It shows good reliability (Cronbach's α = 0.93) and validity, and it is a good predictor of depression and low quality of life (McCracken et al., 2014).
• Chronic Pain Acceptance Questionnaire (CPAQ; McCracken et al., 2004), Spanish version (Rodero et al., 2010). The CPAQ assesses acceptance of pain in patients suffering from a chronic pain condition. It consists of 20 items rated on a Likert-type scale (0: never true – 6: always true). It shows high reliability in FM patients (Cronbach's α = 0.83), including its test-retest reliability (intraclass correlation = 0.83). CPAQ scores have been negatively associated with pain, negative affect, and catastrophizing, and positively with quality of life (Rodero et al., 2010).
• Psychological Inflexibility in Pain Scale (PIPS, Wicksell et al., 2008, Wicksell et al., 2010), Spanish validation (Rodero et al., 2013). It consists of 12 items rated on a Likert-type scale (1: never true – 7: always true). It assesses the level of pain-related avoidance and cognitive fusion. It has been validated with FM patients, showing excellent internal consistency (Cronbach's α = 0.90) and test-retest reliability (intraclass correlation = 0.97). Scores are positively associated with anxiety and depression, and modestly with pain intensity, as well as negatively with pain acceptance and psychosocial functioning (Rodero et al., 2013).

### 2.6 Experiential sampling

Experiential Sampling (ES) was conducted through short Google Forms online surveys (with survey links submitted through a private WhatsApp group) administered during six weeks pre- and six weeks post-intervention. Three surveys were sent to participants every week (hence totaling 18 ES assessments per participant both pre- and post-intervention), randomizing both day and time of each submission. Randomization was carried out using the Excel function “randbetween” with the following parameters: (a) the first weekly survey had to be completed either on Monday or Tuesday, the second one either on Wednesday or Thursday, and the third one either on Friday or Saturday; (b) 40 % of the surveys would be completed in the morning timeframe (10 am to 2 pm), another 40 % in the afternoon-evening timeframe (4 pm to 8 pm), and the remaining 20 % in the noon timeframe (hours culturally considered noon in Spain: 2 pm to 4 pm). Sundays were left survey-free, since this would be the day for therapy sessions. In addition, surveys were never sent on public holidays. Participants were asked to complete surveys as quickly as they noticed the incoming Google Forms link sent to their smartphones. Participants were not aware of the moment (day and hour) in which surveys would be sent.

All ES surveys consisted of ten Likert-type (1–7) items. Three items enquired about outcomes: pain intensity (item 1), emotional discomfort (item 5), and satisfaction with actions (item 9). Seven items enquired about underlying PF-related processes, three regarding pain, three regarding emotional discomfort, and one regarding committed actions in general. The three pain-related items and the three items on emotional discomfort covered psychological inflexibility as a deficit in each of the three dyadic PF processes (Hayes et al., 2011): lack of openness/presence of avoidance (items 2 and 6), inaction/lack of engagement (items 3 and 7), and lack of present moment awareness/presence of rumination (items 4 and 8). The last item covered general engagement in valued actions (10). ES surveys also included three dichotomous questions (Yes/No) about medication intake (analgesics, antidepressants and anxiolytics) in the last 24 h (see Table 1 for the full list of items in the ES form). The estimated time to complete each survey was around three minutes.

**Table 1:** Questions of the experiential sampling surveys.

Experiential sampling survey
ES-Q1. Mark from 1 to 7 the intensity of pain you have right now. NONE (1) - MAXIMUM PAIN (7) ES-Q2. In the last hour, have you tried to eliminate or reduce pain? NOT AT ALL (1) - ALL THE TIME (7) ES-Q3. In the last hour, do you feel that pain has prevented you from doing things that are important to you? NOT AT ALL (1) - TOTALLY (7) ES-Q4. In the last hour, how long have you spent thinking about pain? NOT AT ALL (1) - ALL THE TIME (7) ES-Q5. Mark from 1 to 7 the level of emotional discomfort you have right now. NONE (1) – MAXIMUM EMOTIONAL DISCOMFORT (7) ES-Q6. In the last hour, have you tried to eliminate or reduce emotional discomfort? NOT AT ALL (1) - ALL THE TIME (7) ES-Q7. In the last hour, do you feel like that emotional discomfort has prevented you from doing things that are important to you? NOT AT ALL (1) - TOTALLY (7) ES-Q8. In the last hour, how long have you spent thinking about how bad you feel? NOT AT ALL (1) - ALL THE TIME (7) ES-Q9. Are you satisfied with the actions you have undertaken in the last hour? NOT AT ALL (1) - TOTALLY SATISFIED (7) ES-Q10. In the last hour, have you done what was important to you?? NOT AT ALL (1) - TOTALLY (7) ES-Control 1. I have taken analgesics for pain in the last 24 h. YES/NO ES-Control 2. I have taken antidepressants in the last 24 h. YES/NO ES-Control 3. I have taken anxiolytics in the last 24 h. YES/NO

*Note*. ES = Experiential Sampling, Q = Question.

### 2.7 ACT intervention

The intervention was an online version of ACT developed for this study. It consisted of five weekly 105-minute online group Google Meet video-meeting sessions with the therapist and all participants. The first author (P.d.l.C.), a postdoctoral researcher with extensive experience on psychophysiological assessment of FM and other chronic pain syndromes, conducted the intervention under weekly supervision of the second and third authors (M.R.V. and M.H.L.), registered healthcare psychologists with experience in ACT delivery and training.

The intervention was aimed to enhance the FM patients' openness to experiencing pain and associated emotional discomfort in a centered, conscious manner, choosing to behave consistently with personal values, in order to live a meaningful life even with the constant presence of pain and other chronic symptoms. Throughout the different sessions the participants' pain experience was validated and functionally analyzed, examining the workability of attempts to control pain and other aversive private events, and the impact these attempts were having on their lives. Since participants perceived their pain and emotional discomfort as barriers for a valued, meaningful life, the therapeutic work promoted cognitive defusion, present-moment awareness, and the development of a perspective of the self as hierarchically over these private events (see C. Luciano et al., 2009; Törneke et al., 2016). Values clarification work helped participants identify and undertake specific actions leading to a more meaningful life, and they were actively encouraged to commit to these actions. Table 2 displays the goals addressed in each clinical session. More detailed information on the multiple metaphors and experiential exercises used along the intervention can be requested to the corresponding author.

**Table 2:** ACT intervention session goals.

	Goals
Session #*1*	• To establish ground rules for online group functioning. • Building ACT-consistent therapeutic relationship. • Validation of pain experience and related-symptoms. • Functional analysis of experiential avoidance/inflexibility pattern. • To create a context for change: reframing purpose of therapy (“having a bigger life”).
Session #*2*	• To analyze workability of control based on their experience. • To generate creative hopelessness. • To notice psychological barriers and values are not confronted. • To introduce defusion regarding private events perceived as barriers. • To present self as hierarchically over private events.
Session #*3*	• Contacting the present moment (mindfulness). • Practice of cognitive defusion skills. • Practice framing self in a hierarchical relation with private events. • Values clarification and commitment with valued actions.
Session #*4*	• Recapitulation of prior work on functional analysis and therapy goals. • Further mindfulness practice. • Further practice of cognitive defusion skills. • Further practice of framing self in a hierarchical relation with private events.
Session #*5*	• Further mindfulness practice. • Further practice of cognitive defusion skills. • Further practice of framing self in a hierarchical relation with private events. • Normalization of pain and discomfort and relapse prevention. • Strengthening commitment with valued actions.

### 2.8 Procedure

All of the procedures in this study were approved by the Ethics Review Board of the University of Jaén through the MPGS Ethics Commission (2020/21), according to the ethical principles for medical research in human beings of the Declaration of Helsinki of the World Medicine Association (World Medical Association, 2013).

Potential participants were contacted by phone to inform them about the possibility of participating in the study. Upon verification of inclusion and exclusion criteria, participants read and signed an online statement of informed consent, and were scheduled for a second appointment by phone.

#### 2.8.1 Phase I. Pre-intervention assessment and ES

In the second phone call (approximate duration, 45 min) each participant was individually interviewed for the collection of basic sociodemographic and clinical information, as well as for the pre-intervention administration of the abovementioned questionnaires. All participants were interviewed in two consecutive days. The last minutes of each call were dedicated to arranging several basic aspects of participation (best day and time for online sessions, checking that the participant had an active Google account, etc.) as well as to briefly explaining the ES surveys and verbally collecting participant authorization to be included in a WhatsApp group for the delivery of links to ES surveys. Participants were explicitly informed that the WhatsApp group was exclusively meant for survey access and therapist announcements, and not for contact with the therapist or other group members. During the subsequent 6 weeks, pre-intervention ES was performed through three weekly surveys as previously described (obtaining 18 pre-intervention ES assessments). In addition, participants received a weekly reminder of the time remaining for the first online group session. The last week before intervention, participants received instructions for the installation of the Google Meet App. Individual phone assistance was provided for this matter if necessary.

#### 2.8.2 Phase II: Online group ACT intervention

Five weekly sessions (105 min. duration each) were held on Sundays, as described above in “ACT intervention”. Each clinical session consisted of: initial presentation (or participatory summary on the previous session), review of experiences after practicing the proposed activities at home, presentation of metaphors and practice of experiential exercises, scheduling of practice activities at home, reflections, and resolution of doubts and queries.

#### 2.8.3 Phase III. Post-intervention assessment and 6-month Follow-up

Post-intervention individual interviews were conducted by phone during the two days immediately after the fifth and last online group session. The phone call consisted of the administration of the same questionnaires used during pre-intervention (approx. 45 min). Then, participants received again three weekly ES surveys for another six weeks (getting 18 post-intervention ES assessments). Six months after treatment, participants underwent a final phone-delivered assessment with the same questionnaires administered pre- and post-intervention.

### 2.9 Statistical analysis

All statistical analyses were performed with *IBM SPSS Statistics for Windows, Version 19.0* (IBM Corp., Armonk, NY., USA). According to the Shapiro-Wilk test, there was no deviation from normality for any of the different variables (all *p*s > 0.1).

To determine the feasibility of our intervention, the following indices based on updated guidelines (e.g., Gadke et al., 2021; Pearson et al., 2020) were calculated: Adoption rate (individuals who agreed to participate in the study out of those who expressed interest in participating), Retention rate (participants who completed the study out of those who agreed to participate), Clinical Adherence (proportion of participants attending sessions), ES Completion rate (proportion of completed ES surveys), and Acceptability (level of satisfaction both with the intervention's contents and with its implementation, assessed through a single-selection question with the options: “dissatisfied”, “little satisfied”, “moderately satisfied”, “Satisfied”, or “Very satisfied”).

In order to examine pre-intervention linear trends of the clinical outcome variables collected by ES, a simple linear regression analysis was carried out individually for each participant. This would allow us to discard from further analyses any cases showing clinical improvement before intervention. Pre-post and pre-follow-up group differences for questionnaire-based outcome and process variables were examined by Wilcoxon Z test. Hedges g was calculated to estimate the effect size of changes after intervention at a group level. Regarding ES variables, pre-post group and individual comparisons were examined by Receiver Operating Characteristic (ROC) analyses, using the Area under the ROC curve (AUC) to obtain the Non-overlap of All Pairs (NAP) index and its 95 % Confidence Interval. NAP reports the proportion of all pairs of one ES measurement across phases in which the post-intervention measurement entails an improvement upon the pre-intervention measurement. NAP was calculated for each ES variable and participant for the pre-post individual comparisons, whilst for the pre-post group comparisons NAP was calculated using an average of all participants for each one of the ES measurements in each variable. NAP was converted to a percent scale (NAP_0__–__100_) in order to facilitate its interpretation [NAP_0__–__100_ = (NAP/0.5–1) x 100]. Thus, NAP = 0.5 would become NAP_0__–__100_ = 0 %, pointing to null improvement (i.e., as a random outcome); whereas NAP = 1 would become NAP_0__–__100_ = 100 %, indicating total improvement after intervention (i.e., all ES post-intervention measurements would improve upon all ES pre-intervention measurements). Effect sizes were interpreted based on Cohen (1992) for Hedges g, and on Parker and Vannest (2009) for NAP_0__–__100_. Additionally, parallel analyses were carried out for pre-post comparisons including medication intake (analgesics, antidepressants and anxiolytics) as covariates in order to rule out the influence of medication on results. All findings remained the same.

Following Lavefjord et al.'s (2021) recommendation of adopting an idiographic framework, we estimated the clinical significance of change individually for all outcome and process variables, both questionnaire-based and ES-based. Accordingly, we established that “Clinical Improvement or Worsening” would be determined by post-intervention changes of at least ±1 SD from pre-intervention levels in a functional or dysfunctional direction, respectively. Considering that post-intervention data were only compared to pre-intervention data from this same study (and not to external benchmarking scores), a criterion of ±0.5 SD (Norman et al., 2004) used in similar studies with ES measurements (e.g. Chisari et al., 2022) might have been too sensitive (qualifying potential random variation as significant change). In turn, a criterion of ±2 SD (McGlinchey et al., 2002) might have been too strict, since it could yield cutoff scores well under/over the average non-clinical population score or even out-of-range scores for some questionnaire-based measures. Anyway, for illustrative purposes, changes based on ±2 SD were also reported. Besides, for ES variables, post-intervention trends had to be either non-significant (indicating maintenance of post-intervention effects) or significant but pointing to a clinical improvement.

In relation to the second goal of the study, we carried out multilevel regression analyses based on restricted maximum likelihood estimates, in order to find the predictive model with greatest power to account for outcome ES variables, using as predictors both process ES variables and questionnaire-based outcome variables. These analyses examined potential variables to account for the greatest possible proportion of within- and between-case variance of post-intervention ES outcome variables. First and second level predictors were distinguished. At level 1 (within-case), all measurements of each participant were used for each variable, thus each one had 162 data (18 post-intervention measurements x 9 participants). At level 2 (between-cases), the outcome questionnaire scores of each participant were taken and ES variables were adapted to multilevel analysis by averaging the ratings for each ES question by participant. This took into account both the effect of ES process measurements, linked to the momentary context of each measurement and participant (within-case level), and questionnaire-based outcome scores, presumably more stable measures (between-case level). At this point, it is required to note that all of these multilevel analyses were implemented using the post-intervention variables.

First, Null Models were estimated using random effects ANOVA in which *Cases* (the grouping variable consisting of numerical values from 1 to 9 identifying each participant) was implemented as “subject variable”. Estimates of fixed and random effects parameters, and covariances of level 1 and 2 for each dependent variable (ES outcome variables: pain intensity [Q1], emotional discomfort [Q5] or satisfaction with actions [Q9]) were obtained without entering any covariate. Subsequently, a level 2 covariate (2Lv Model) and one more level 1 covariate (2 + 1Lv Model) were added to null model. Finally, a model implementing a level 1 covariate and adding both fixed and the random components was estimated by Random Coefficients regression (1Lv-RC Model). The 1Lv-RC models were performed indicating “Unstructure” as the variance structure option, since in this model independence between the parameters is not assumed and data covariation structure is a priori unknown.

Accordingly, Null Models reported data on the within- and between-*Cases* variability of ES measurements for each dependent variable (which could be both reduced by adding covariates of level 1 and 2) considering its intercept as fixed effects variable and the grouping variable (*Cases)* as random effects variable. Then, if variance for *Cases* was significant, it meant that there was an effect of higher level (level 2) on the dependent variable (level 1), and therefore multilevel modeling was necessary. Consequently, 2Lv Models were performed. In turn, significant differences in the residuals (level 1; within-*Cases* variance of ES measurements) were found, thus 2 + 1Lv Models were also conducted. Finally, considering both the differences in within-*Cases* variance of ES measurements for each dependent variable, and that these might randomly vary in each *Case*, 1Lv-RC Models were performed adding a level 1 covariate as random effects to check if greater variability could be explained. Since there was no 1Lv-RC model whose composition of variances revealed the need to implement one more covariate to explain the differences in means and slopes of association between level 1 and dependent covariates, it was unnecessary to estimate any random intercept and slope regression model.

Comparisons of global adjustment for the different models were estimated by -2 Log Likelihood or *devianza* (-2LL) statistic. The less *devianza* of one model compared to another, the better its explanatory fit. It is known that the difference between the *devianza* of two models is distributed according to a chi-square distribution with as many degrees of freedom as the number of parameters in which compared models differ (McCullagh and Nelder, 2019). Thus, the gain of one model regarding another was estimated applying this to the difference of *devianza* in all pairs of compared models. Taking into account the small sample size and the potential multicollinearity of predictors, the best within- and between-case predictors were selected for each outcome ES variable before conducting the multilevel regressions. Multiple stepwise linear regression analyses were performed in order to find the predictor that significantly accounted for the largest proportion of within- and between-variance for each ES outcome variable (i.e. the best predictor amongst [a] all ES process variables, and amongst [b] all questionnaire-based outcome variables).

Finally, in order to address the third goal of the study, we calculated Pearson correlations between the average of each post-intervention ES process variable and the most significant questionnaire-based outcome variable (FIQ) at 6-month follow-up. Additionally, in order to determine the best predictor/s of FIQ at 6-month follow-up. we conducted a stepwise linear regression entering as predictors both the significantly-correlated ES process variables and the FIQ itself at post-intervention.

## 3 Results

### 3.1 Feasibility (Aim 1A)

The adoption rate was 75 %, with 12 FM patients accepting to take part in the study out of the 16 who initially showed interest in participating. The retention rate was 75 %, with three participants dropping out of the study, out of the 12 who accepted participating. The remaining nine participants attended all treatment sessions (100 % Clinical Adherence rate) and completed all pre- and post-intervention ES surveys (100 % ES completion rate). Lastly, acceptability of the intervention was good, with 67 % of the participants reporting to be very satisfied, 22 % reporting to be satisfied, and only 11 % reporting moderate satisfaction with the treatment.

### 3.2 Linear trends of pre-intervention ES outcome variables by participant (Aim 1B)

Only one participant showed a significant linear trend for pain intensity (Q1) [participant #6 (В = 0.472; *t* = 2.142; *p* = 0.048)]. Since this trend entailed a clinical impairment, this participant's data were included in the rest of data analyses.

### 3.3 Pre-post and pre-follow up group comparisons for questionnaire-based variables (Aim 1B)

Table 3 presents pre-post and pre-follow up differences in the average scores of questionnaire-based variables, as well as their effect sizes. Regarding outcome variables, there were significant post-intervention decreases in affective pain, anxiety, and FM impact that were maintained throughout follow-up, with the largest-sized effects for anxiety. For process variables, significant changes were only observed for pain-related variables. While post-intervention decreases in general experiential avoidance and cognitive fusion were not significant, there were a significant increase in pain acceptance and a significant decrease in pain-related psychological inflexibility. These significant changes were maintained throughout follow-up.

**Table 3:** Pre-post and pre-follow up group (n = 9) comparisons for questionnaire-based variables.

Variables	Pre-test Mean ± SD	Post-test Mean ± SD	Follow-up Mean ± SD	*Pre-post*		*Pre-follow up*	
				Wilcoxon Z	Hedges g	Wilcoxon Z	Hedges g
Outcome variables							
Evaluative Pain (MPQ-E)	3.11 ± 0.60	2.89 ± 0.60	3.00 ± 0.87	1.000	0.35 (small)	0.577	0.14 (small)
Affective Pain (MPQ-A)	5.00 ± 2.06	3.89 ± 2.62	3.22 ± 2.28	2.157⁎	0.45 (small)	2.345⁎	0.78 (medium)
Anxiety (HAS)	11.89 ± 4.07	7.56 ± 3.81	7.33 ± 2.87	2.558⁎⁎	1.05 (large)	2.384⁎	1.23 (large)
Depression (HDS)	7.00 ± 3.80	5.67 ± 2.45	5.44 ± 3.21	1.378	0.40 (small)	0.718	0.42 (small)
Fibromyalgia Impact (FIQ)	67.78 ± 18.63	59.67 ± 17.41	53.78 ± 18.39	2.380⁎	0.43 (small)	1.838⁎	0.72 (medium)

Process variables							
Experiential Avoidance (AAQ-II)	29.11 ± 14.03	24.22 ± 11.01	23.11 ± 9.17	0.833	0.37 (small)	1.364	0.48 (small)
Cognitive Fusion (CFQ)	32.11 ± 13.36	25.00 ± 9.82	23.11 ± 9.32	1.248	0.58 (medium)	1.481	0.74 (medium)
Chronic Pain Acceptance (CPAQ)	44.78 ± 19.94	68.44 ± 13.46	91.56 ± 17.76	2.310⁎	1.32 (large)	2.668⁎⁎	2.36 (large)
Psychological Inflexibility (PIPS)	54.44 ± 15.17	38.56 ± 14.24	31.67 ± 17.39	2.310⁎	1.03 (large)	2.547⁎⁎	1.33 (large)

*Note*. Mean ± SD of pre, post and follow-up test scores.

Cohen (1992) effect size guidelines: ≥ 0.20 = small; 0.50 = medium; 0.80 = large.

⁎ ≤0.05.

⁎⁎ ≤0.01.

### 3.4 Clinical significance of the individual changes in questionnaire-based variables (Aim 1B)

Table 4 displays the clinical significance of individual change in each questionnaire-based variable both post-intervention and at the 6-month follow-up. The largest changes in outcome variables were for anxiety, with six participants showing a clinically significant decrease at follow-up. Likewise, four participants showed clinically significant decreases in affective pain and FM impact. For process variables, the most clearly replicated effects were for pain acceptance and pain-related psychological inflexibility, with seven participants in each case showing a clinically significant improvement at follow-up.

**Table 4:** Clinical significance of the changes in questionnaire-based variables by participant.

Outcome variables											Process variables							
Patient	Evaluative pain (MPQ-E)		Affective pain (MPQ-A)		Anxiety (HAS)		Depression (HDS)		Fibromyalgia impact (FIQ)		Experiential avoidance (AAQ-II)		Cognitive fusion (CFQ)		Chronic pain acceptance (CPAQ)		Psych. Inflexibility in pain (PIPS)	
	Pre-post	Pre-followup	Pre-post	Pre-followup	Pre-post	Pre-followup	Pre-post	Pre-followup	Pre-post	Pre-followup	Pre-post	Pre-followup	Pre-post	Pre-followup	Pre-post	Pre-followup	Pre-post	Pre-followup
1	↓	↓		↑						↑						↑		↑
2	↑		↑		↑↑	↑	↑	↑↑	↑	↑	↑↑	↑↑	↑↑	↑↑	↑↑	↑↑	↑	↑
3					↑												↑	↑
4															↑	↑		
5	↑	↑	↑			↑				↑			↑	↑	↑↑	↑↑	↑	↑↑
6		↑		↑	↑	↑↑	↑	↑		↑		↑	↑	↑	↑	↑↑	↑	↑↑
7						↑									↑	↑↑		↑
8			↑	↑	↑	↑		↓							↑	↑↑	↑↑	↑↑
9	↑			↑	↑	↑												

*SD*_*pre*_ *=* Standard Deviation from pre-intervention sample distribution; ↑ = change of ±1*SD*_*pre*_ in functional direction; ↑↑ = change of ±2*SD*_*pre*_ in functional direction; ↓ = change of ±1*SD*_*pr*e_ in dysfunctional direction.

### 3.5 Pre-post group comparisons for ES variables (Aim 1B)

All ES variables showed significant post-intervention changes, except for pain intensity (Q1). Pre-post group comparison outcomes for ES variables are provided in Table 5. Interpretation of effect sizes associated to the changes after intervention can be also found.

**Table 5:** Pre-post group (n = 9) comparisons for average ES variables.

Variables	Pre Mean ± SD	Post Mean ± SD	Pre-Post		
			NAP	95 % CI	NAP _0__–__100_ [%]
Outcome variables					
Pain Intensity (Q1)	5.04 ± 1.11	4.75 ± 1.10	0.66	0.48–0.84	32 % (small)
Emotional Discomfort (Q5)	4.08 ± 1.90	3.31 ± 2.08	0.95⁎	0.89–1.00	90 % (medium)
Satisfaction with actions (Q9)	3.95 ± 1.71	4.48 ± 1.91	0.80⁎	0.65–0.94	59 % (small)

Process variables					
Pain Avoidance (Q2)	3.98 ± 1.85	3.44 ± 2.05	0.83⁎	0.70–0.97	66 % (medium)
Pain-related Inaction (Q3)	4.26 ± 2.05	3.43 ± 1.84	0.90⁎	0.79–1.00	79 % (medium)
Pain Rumination (Q4)	3.99 ± 1.20	3.58 ± 1.44	0.74⁎	0.58–0.91	49 % (small)
Discomfort Avoidance (Q6)	3.38 ± 1.87	3.08 ± 2.31	0.74⁎	0.56–0.91	48 % (small)
Discomfort-related Inaction (Q7)	3.77 ± 2.23	2.99 ± 2.07	0.95⁎	0.89–1.00	90 % (medium)
Discomfort Rumination (Q8)	3.69 ± 1.74	3.03 ± 1.84	0.89⁎	0.78–0.99	77 % (medium)
Valued Actions (Q10)	3.98 ± 1.96	4.52 ± 1.85	0.82⁎	0.67–0.96	63 % (small)

*Note*. Mean ± SD of pre-intervention and post-intervention average ES measurements. 95 % CI = 95 % Confidence interval.

Parker and Vannest (2009) effect size guidelines: 0–65 % = small; 66–92 % = medium; 93–100 % = large.

⁎ ≤0.01.

### 3.6 Pre-post individual comparisons for ES variables (Aim 1B)

The effect sizes for each participant in outcome and process ES variables at post-intervention are provided in Table 6. Regarding outcome variables, the largest effects sizes were found for emotional discomfort (Q5), while only small or no effect sizes were observed for satisfaction with actions (Q9) and pain intensity (Q1). There was large variability in effect sizes across participants for each process variable.

**Table 6:** Effect sizes for pre-post change in outcome and process ES variables by participant.

Variables	Participants	NAP	95%CI	N_0__–__100_	NAP	95%CI	N_0__–__100_	NAP	95%CI	N_0__–__100_	NAP	95%CI	N_0__–__100_
Pain-related variables (Q1 – Q4)		Pain Intensity (Q1)			Pain Avoidance (Q2)			Pain-related inaction (Q3)			Pain Rumination (Q4)		
	1	0.597	0.407–0.788	19 %	0.367	0.182–0.552	-27 %	0.914	0.803–1.000	83 %	0.750	0.589–0.911	50 %
	2	0.437	0.245–0.629	−13 %	0.437	0.247–0.626	−13 %	0.519	0.325–0.712	4 %	0.474	0.280–0.668	−5 %
	*3*	0.688	0.510–0.867	38 %	0.887	0.779–0.996	77 %	0.639	0.455–0.823	28 %	0.727	0.563–0.890	45 %
	4	0.517	0.325–0.709	3 %	0.338	0.159–0.517	−32 %	0.620	0.432–0.808	24 %	0.378	0.194–0.563	−24 %
	5	0.603	0.417–0.789	21 %	0.889	0.771–1.000	78 %	0.864	0.734–0.994	73 %	0.824	0.686–0.963	65 %
	6	0.670	0.489–0.851	34 %	0.306	0.129–0.482	−39 %	0.736	0.564–0.908	47 %	0.500	0.305–0.695	0 %
	7	0.625	0.441–0.809	25 %	0.708	0.538–0.878	−42 %	0.679	0.505–0.853	36 %	0.617	0.428–0.806	23 %
	8	0.515	0.323–0.708	3 %	0.557	0.367–0.747	11 %	0.477	0.286–0.668	−5 %	0.508	0.315–0.701	2 %
	9	0.596	0.409–0.783	19 %	0.722	0.551–0.893	44 %	0.815	0.674–0.955	63 %	0.813	0.666–0.960	63 %

		Emotional Discomfort (Q5)			Discomfort Avoidance (Q6)			Discomfort-rel. inaction (Q7)			Discomfort Rumination (Q8)		
Discomfort-related variables (Q5 – Q8)	1	0.878	0.767–0.989	76 %	0.519	0.323–0.714	4 %	0.932	0.854–1.000	86 %	0.770	0.615–0.925	54 %
	2	0.806	0.663–0.949	61 %	0.606	0.420–0.793	21 %	0.639	0.455–0.823	28 %	0.776	0.623–0.930	55 %
	3	0.585	0.397–0.773	17 %	0.583	0.395–0.772	17 %	0.583	0.395–0.772	17 %	0.616	0.430–0.802	23 %
	4	0.460	0.269–0.651	−8 %	0.387	0.201–0.573	−23 %	0.593	0.403–0.782	19 %	0.415	0.227–0.603	17 %
	5	0.849	0.713–0.985	70 %	0.750	0.584–0.916	50 %	0.750	0.584–0.916	50 %	0.699	0.524–0.874	40 %
	6	0.392	0.202–0.582	−22 %	0.250	0.084–0.416	−50 %	0.667	0.486–0.847	33 %	0.500	0.309–0.691	0 %
	7	0.446	0.256–0.636	−11 %	0.444	0.254–0.634	−11 %	0.444	0.254–0.634	−11 %	0.417	0.228–0.605	−17 %
	8	0.576	0.386–0.765	15 %	0.543	0.352–0.734	9 %	0.602	0.414–0.790	20 %	0.585	0.395–0.774	17 %
	9	0.756	0.597–0.915	51 %	0.617	0.429–0.805	23 %	0.701	0.526–0.875	40 %	0.816	0.671–0.962	63 %

		Satisfaction with Actions (Q9)			Valued Actions (Q10)								
Action-related variables (Q9 – Q10)	1	0.779	0.628–0.931	56 %	0.870	0.750–0.990	74 %						
	2	0.534	0.341–0.727	7 %	0.512	0.312–0.713	2 %						
	3	0.560	0.370–0.750	12 %	0.583	0.395–0.772	17 %						
	4	0.455	0.257–0.653	−9 %	0.503	0.306–0.700	1 %						
	5	0.755	0.592–0.918	51 %	0.602	0.410–0.794	20 %						
	6	0.529	0.338–0.721	6 %	0.617	0.431–0.804	23 %						
	7	0.704	0.533–0.875	41 %	0.602	0.414–0.790	20 %						
	8	0.426	0.236–0.616	−15 %	0.556	0.365–0.746	11 %						
	9	0.551	0.361–0.741	10 %	0.579	0.391–0.767	16 %						

95 % CI = 95 % Confidence interval. Parker and Vannest (2009) effect size guidelines: 0–65 % = small; 66–92 % = medium; 93–100 % = large.

**Fig. 1:**
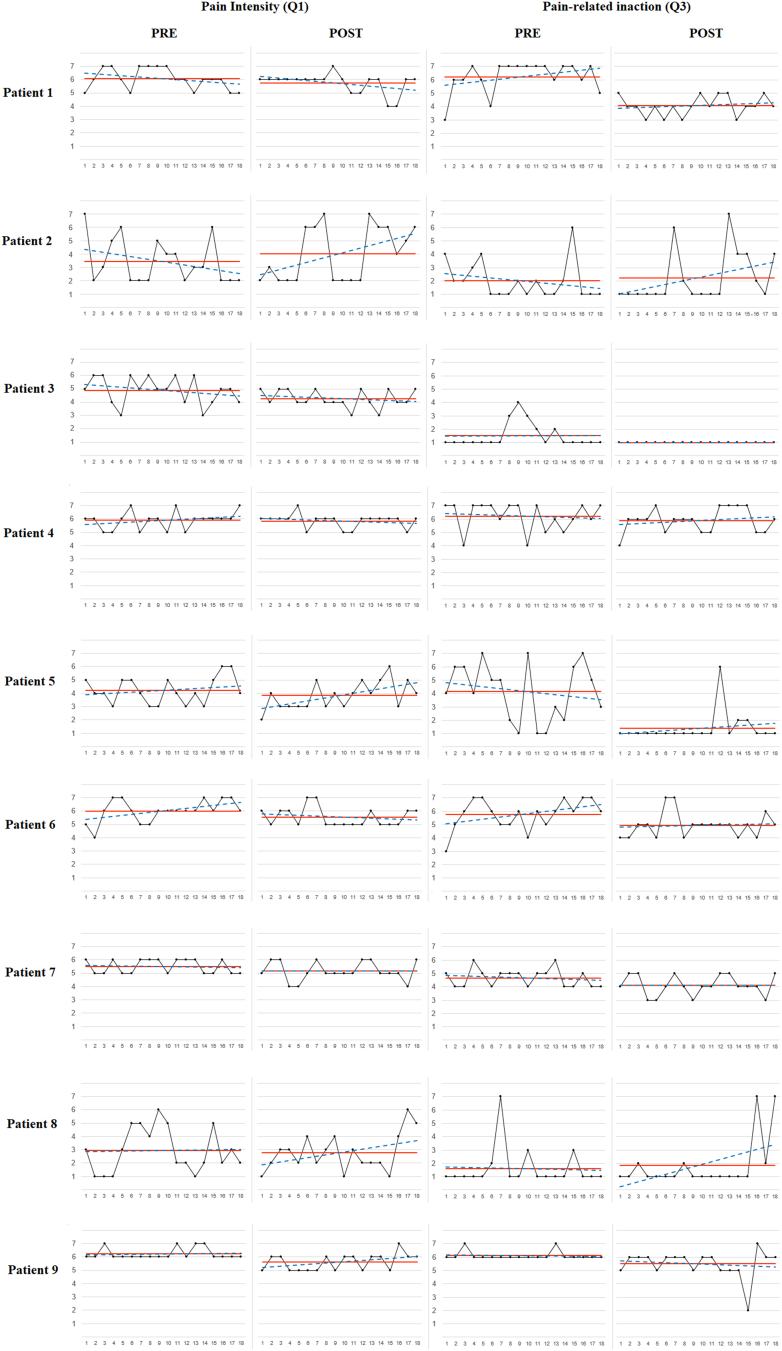
*Note.* Raw measurements, average line and linear trend for pain intensity (Q1) and pain-related inaction (Q3) at pre- vs. post-intervention periods by participant.

**Fig. 2:**
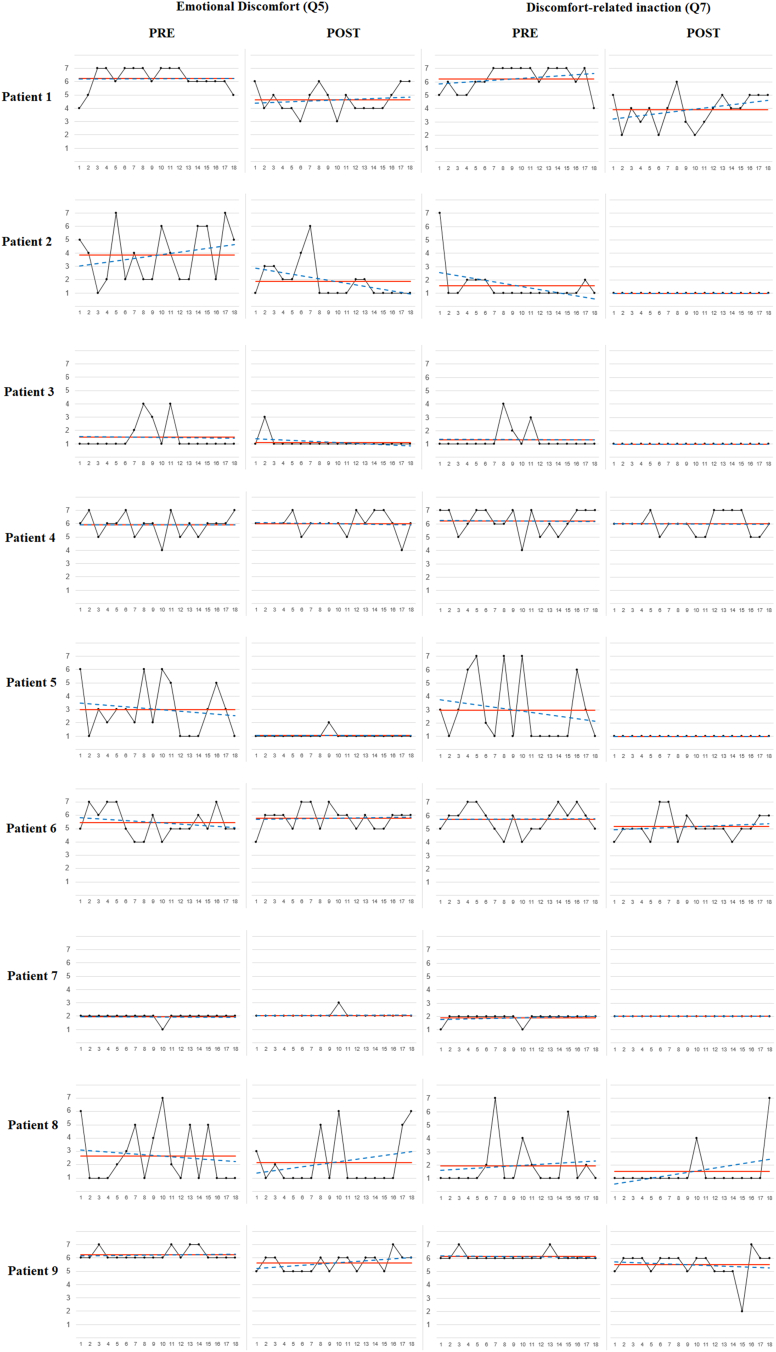
*Note.* Raw measurements, average line and linear trend for emotional discomfort (Q5) and discomfort-related inaction (Q7) at pre- vs. post-intervention periods by participant.

**Fig. 3:**
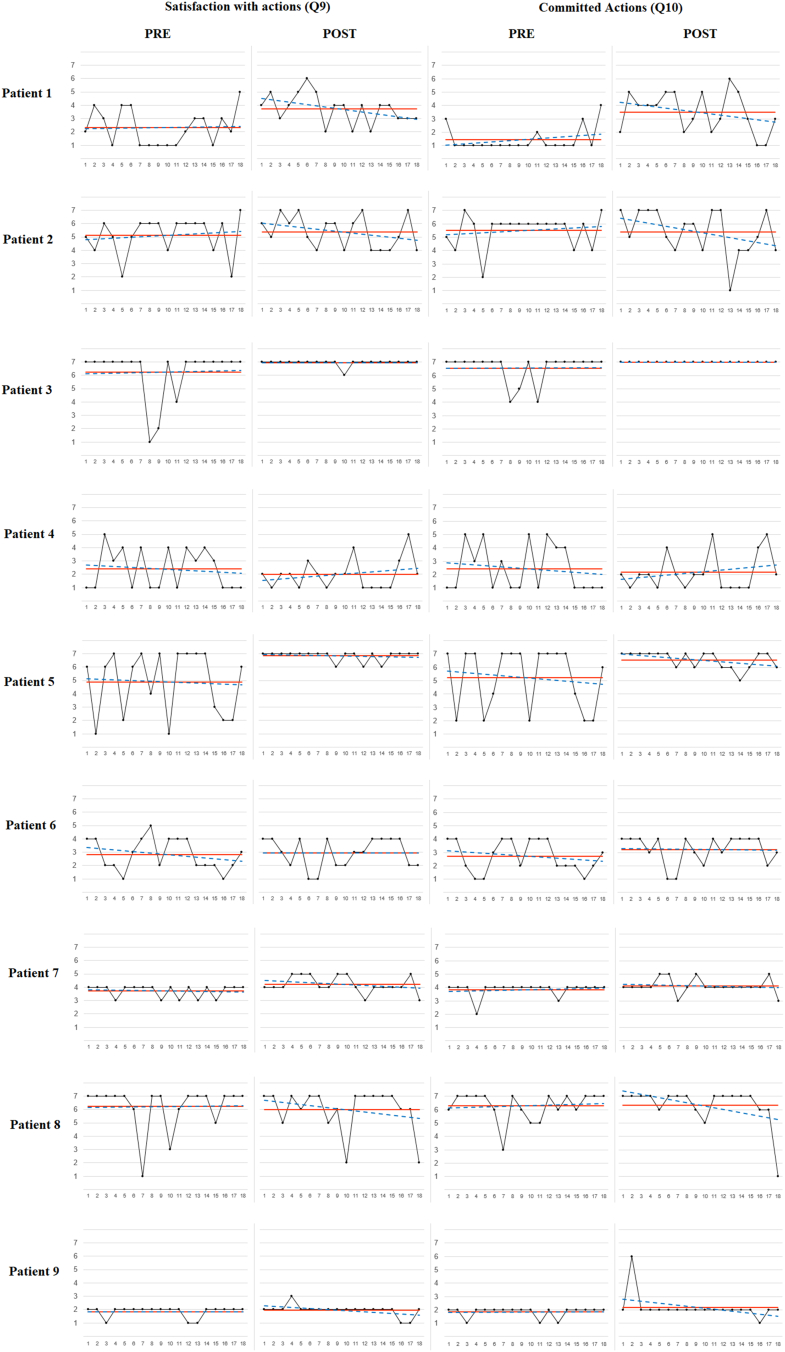
*Note.* Raw measurements, average line and linear trend for satisfaction with actions (Q9) and valued actions (Q10) at pre- vs. post-intervention periods by participant.

For illustrative purposes, Fig. 1, Fig. 2, Fig. 3 display the raw measurements, average and linear trend for the three ES outcome variables at pre- vs. post-intervention periods for each participant. Additionally, the most strongly correlated-ES process variable was included in each one of the three figures. All participants showed nil or improving trends post-intervention for the three outcome variables, except for two participants presenting a significant positive linear trend in pain intensity (Q1) [participant #2 (В = 0.471; *t* = 2.137; *p* = 0.048); participant #5 (В = 0.576; *t* = 2.816; *p* = 0.012)], and one participant displaying a significant negative linear trend in satisfaction with actions (Q9) [participant #5 (В = −0.516; *t* = −2.411; *p* = 0.028)]. Overall, post-intervention effects were maintained throughout the 6-week post-intervention ES measurement period for most participants.

### 3.7 Clinical significance individual changes in ES variables (Aim 1B)

Table 7 shows the clinical significance of individual change in each ES variable after intervention. The largest clinically significant changes in outcome variables were for emotional discomfort (Q5) and satisfaction with actions (Q9), with three participants showing a clinically significant improvement. Only one participant showed a clinically significant decrease in pain intensity (Q1), whereas another presented a clinically significant increase. For process variables, the largest clinically significant changes were for pain avoidance (Q2) and discomfort-related inaction (Q7), with five and four participants showing a clinically significant improvement, respectively. In turn, pain rumination (Q4) and discomfort avoidance (Q6) were the process variables presenting less clinically significant change across participants, with two participants showing clinically significant improvement in both cases.

**Table 7:** Clinical significance of pre-post changes in ES variables by participant.

Participant	Pain Intensity (Q1)	Pain Avoidance (Q2)	Pain-related Inaction (Q3)	Pain Rumination (Q4)	Emotional Discomfort (Q5)	Discomfort Avoidance (Q6)	Discomfort-related Inaction (Q7)	Discomfort Rumination (Q8)	Satisfaction with actions (Q9)	Valued Actions (Q10)
1			↑↑		↑↑		↑↑	↑		↑
2	↓				↑		↑	↑		
3		↑↑	↑	↑		↑	↑		↑	↑
4										
5		↑	↑		↑	↑	↑		↑	
6										
7		↑							↑	
8										
9		↑↑		↑↑			↑↑	↑↑		↑

*SD*_*pre*_ *=* Standard Deviation from pre-intervention sample distribution; ↑ = change of ±1*SD*_*pre*_ in functional direction; ↑↑ = change of ±2*SD*_*pre*_ in functional direction; ↓ = change of ±1*SD*_*pr*e_ in dysfunctional direction.

### 3.8 Multilevel models for post-intervention ES outcome variables (Aim 2)

Firstly, stepwise multiple regression analyses revealed which would be the best within- and between-case predictors to estimate multilevel models of post-intervention ES outcome variables. Regarding within-case predictors (post-intervention ES process variables), pain-related inaction (Q3) emerged as the most significant predictor of pain intensity, discomfort-related inaction (Q7) as the most significant predictor of emotional discomfort (Q5), and valued actions (Q10) was selected as predictor of satisfaction with actions (Q9) (being the only available predictor for this ES outcome variable) (all Вs ≥ |0.857|; all *p*s ≤ 0.003). As for between-case predictors (post-intervention questionnaire-based outcome variables), the first regression analysis pointed to the Impact of FM (FIQ) as the most significant predictor of pain intensity (Q1), emotional discomfort (Q5), and satisfaction with actions (Q9) (all Вs ≥ |0.737|; all *p*s ≤ 0.023).

Multilevel models were performed to explain the variance of the post-intervention ES outcome variables pain intensity (Q1), emotional discomfort (Q5) and satisfaction with actions (Q9). Devianzas (-2LL) and estimates of fixed parameters of each model can be found in Table 8, and estimates of covariance parameters in Table 9.

**Table 8:** Devianzas and estimates of fixed parameters of the models for ES outcome variables post-intervention (*n* = 9).

Variable	Model	-2LL (df)	Parameter	Value	Error	t	*p*
Intensity Pain (Q1)	Null	501.046 (3)	Intercept	4.78	0.37	13.01	<0.001⁎⁎
	2Lv	501.159 (4)	Intercept FIQ (level 2)	1.98 0.05	0.99 0.02	1.99 2.92	0.087 0.022⁎
	2+1Lv	438.143 (5)	Intercept FIQ (level 2) Q3 (level 1)	3.36 -0.01 0.54	0.74 0.01 0.06	4.55 -0.55 9.10	0.002⁎⁎ 0.591 <0.001⁎⁎
	1Lv-RC	429.149 (6)	Intercept Q3 (level 1)	3.10 0.49	0.38 0.08	8.19 6.31	<0.001⁎⁎ 0.001⁎⁎
Emotional Discomfort (Q5)	Null	485.317 (3)	Intercept	3.35	0.70	4.77	0.001⁎⁎
	2Lv	474.818 (4)	Intercept FIQ (level 2)	−3.42 0.11	0.98 0.02	−3.51 7.20	0.01⁎ <0.001⁎⁎
	2+1Lv	400.954 (5)	Intercept FIQ (level 2) Q7 (level 1)	-0.87 0.04 0.70	0.49 0.01 0.07	-1.78 3.51 10.55	0.105 0.002⁎⁎ <0.001⁎⁎
	1Lv-RC	398.843 (6)	Intercept Q7 (level 1)	1.03 0.86	0.40 0.08	2.59 10.83	0.44 <0.001⁎
Satisfaction with actions (Q9)	Null	488.033 (3)	Intercept	4.45	0.65	6.83	<0.001⁎⁎
	2Lv	474.143 (4)	Intercept FIQ (level 2)	10.91 -0.11	0.70 0.11	15.50 -9.51	<0.001⁎⁎ <0.001⁎⁎
	2+1Lv	344.806 (5)	Intercept FIQ (level 2) Q10 (level 1)	4.01 -0.04 0.65	0.56 0.01 0.04	7.20 -6.22 14.70	<0.001⁎⁎ <0.001⁎⁎ <0.001⁎
	1Lv-RC	337.888 (6)	Intercept Q10 (level 1)	1.19 0.68	0.40 0.10	3.00 7.03	0.022⁎ <0.001⁎⁎

*Note*. Null model = model without any covariate; 2Lv model = null model entering a level 2 covariate; 2 + 1Lv model = 2Lv model entering a level 1 covariate; 1Lv-RC model = null model entering a level 1 covariate with fixed and random components; FIQ = Fibromyalgia Impact Questionnaire score; Q3 = Pain-related Inaction; Q7 = Discomfort-related Inaction; Q10 = Valued Actions.

⁎ <0.05.

⁎⁎ <0.01

**Table 9:** Estimates of covariance parameters of the models for ES outcome variables post-intervention (*n* = 9).

Variable	Model	Parameter	Value	Error	Wald Z	*p*
Pain Intensity (Q1)	Null	Residuals (level 1) Cases (level 2)	1.10 1.16	0.13 0.61	8.75 1.90	<0.001⁎⁎ 0.057
	2Lv	Residuals (level 1) Cases (level 2)	1.10 0.57	0.13 0.34	8.75 1.69	<0.001⁎⁎ 0.091
	2 + 1Lv	residuals (level 1) Cases (level 2)	0.74 0.29	0.08 0.18	8.71 1.63	<0.001⁎⁎ 0.103
	1Lv-RC	Residuals (level 1) Cases (level 2) Cases – Slopes Slopes	0.70 0.75 -0.11 0.02	0.08 0.66 0.13 0.03	8.45 1.14 -0.84 0.85	<0.001⁎⁎ 0.254 0.3990 0.395
Emotional Discomfort (Q5)	Null	Residuals (level 1) Cases (level 2)	0.93 4.39	0.11 2.22	8.75 1.98	<0.001⁎⁎ 0.048⁎
	2Lv	Residuals (level 1) Cases (level 2)	0.93 0.55	0.11 0.32	8.75 1.71	<0.001⁎⁎ 0.087
	2+1Lv	Residuals (level 1) Cases (level 2)	0.61 0.08	0.07 0.06	8.73 1.31	<0.001⁎⁎ 0.190
	1Lv-RC	Residuals (level 1) Cases (level 2) Cases – Slopes Slopes	0.59 1.15 -0.23 0.04	0.07 0.83 0.18 0.03	8.61 1.39 -1.25 1.07	<0.001⁎⁎ 0.163 0.211 0.283
Satisfaction with actions (Q9)	Null	Residuals (level 1) Cases (level 2)	0.95 3.77	0.11 1.91	8.75 1.97	<0.001⁎⁎ 0.049⁎
	2Lv	Residuals (level 1) Cases (level 2)	0.95 0.26	0.11 0.17	8.75 1.56	<0.001⁎⁎ 0.120
	2+1Lv	Residuals (level 1) Cases (level 2)	0.43 0.03	0.05 0.03	8.71 1.06	<0.001⁎⁎ 0.289
	1Lv-RC	Residuals (level 1) Cases (level 2) Cases – Slopes Slopes	0.36 0.92 -0.18 0.07	0.04 0.65 0.14 0.04	8.45 1.41 -1.28 1.56	<0.001⁎⁎ 0.157 0.202 0.119

*Note*. Null model = model without any covariate; 2Lv model = null model entering a level 2 covariate; 2 + 1Lv model = 2Lv model entering a level 1 covariate; 1Lv-RC model = null model entering a level 1 covariate with fixed and random components.

⁎ <0.05.

⁎⁎ <0.01.

### 3.9 Multilevel models for Pain Intensity (Q1). (Aim 2)

Regarding pain intensity (Q1), the devianza of the 1LV-RC model (429.149; the lowest devianza model) was significantly lower (χ2 = 8.99; df = 1; *p* = 0.003) than that of the 2 + 1Lv model (438.143; the one with the second-lowest devianza), pointing to the 1Lv-RC model, with pain-related inaction (Q3, level 1) as the only predictor variable, as the best fit model to account for pain intensity (Q1). Inclusion of pain-related inaction in the 1Lv-RC model reduced the variance of level 2 by 40 % compared to the null model ([1.90–1.14]/1.90).

### 3.10 Multilevel models for Emotional Discomfort (Q5). (Aim 2)

In regard to emotional discomfort (Q5), the devianza of the 1LV-RC model (398.843; the lowest devianza model) was lower than that of the 2 + 1Lv model (400.954; the one with the second-lowest devianza), but this difference was not statistically significant (χ^2^ = 2.11; df = 1; *p* = 0.146). However, compared to the 2Lv model (438.143), the 1LV-RC model showed a significantly better fit (χ2 = 39.33; df = 2; *p* ≤0.001). Thus, 1Lv-RC, with emotional discomfort-related inaction (Q7, level 1) as the only predictor variable, appeared as the best fit model to account for emotional discomfort (Q5). Inclusion of emotional discomfort-related inaction (Q7) in the 1Lv-RC model reduced level 2 variance by 30 % compared to the null model ([1.98–1.39]/1.98).

### 3.11 Multilevel models for Satisfaction with actions (Q9). (Aim 2)

Finally, for satisfaction with actions (Q9), the devianza of the 1Lv-RC model (337.888, the lowest devianza model) was significantly lower (χ2 = 6.92; df = 1; *p* = 0.009) than that of the 2 + 1Lv model (344.806, the with the second-lowest devianza), pointing to the 1Lv-RC model, with valued actions (Q10, level 1) as the only predictor variable, as the best fit model to account for satisfaction with actions (Q9). Inclusion of valued actions (Q10) in the 1Lv-RC model reduced level 2 variance by 28 % compared to the null model ([1.97–1.41]/1.97).

In addition, for all multilevel models of the three ES outcome variables, level 2 covariances of 2 + 1Lv and 1Lv-RC models presented *p*s > 0.1, ruling out the need to include level 2 explanatory variables using random intercept and slope regression models.

### 3.12 Associations between post-intervention ES process variables and Fibromyalgia Impact at follow-up (Aim 3)

All ES process variable averages for the 6-week post-intervention period significantly correlated with FM impact (FIQ) at the 6-month follow-up (all rs ≥ |0.688|; all *p*s ≤ 0.041), except for pain avoidance (Q2; *r* = 0.610; *p* ≤ 0.081). The stepwise linear regression (entering as predictors both the significantly-correlated ES process variables and the post-intervention FIQ score itself) revealed pain rumination (Q4) as the only significant predictor of FM impact at the 6-month follow-up (В = 0.811; *t* = 3.662; *p* = 0.008).

## 4 Discussion

The present study proved that a brief group online ACT intervention in FM with data collection through smartphone delivered ES was feasible. The intervention was accessible for participants, with 75 % of participants who consented to participate entirely completing it (with 100 % adherence to sessions and ES completion), as well as acceptable, with all treatment completers positively rating the intervention in terms of its contents and implementation. In addition, the intervention was preliminarily effective in producing significant improvement in different aspects relevant to the FM patients' quality of life. Furthermore, the study showed that a contextualized assessment of outcome and process variables through smartphone-delivered ES was easy to implement with widely available, popular online tools, and that it proved useful in providing relevant information (beyond standard questionnaires) for an analysis of the processes of change involved in clinical progress for these patients.

Significant improvements were observed for affective pain, anxiety and biopsychosocial impact of FM after intervention, with these effects holding at the 6-month follow-up. These effects were observed both at the group and individual levels, with most participants reporting clinically significant improvements in these questionnaire-based outcomes. These findings are consistent with those from previous research on ACT interventions for FM patients, both in online (Ljótsson et al., 2014; Simister et al., 2018) and face-to-face formats (J. Luciano et al., 2014; Jensen et al., 2012; Wicksell et al., 2013). Likewise, clinical improvements were observed for ES-based outcomes, with reductions of emotional discomfort and increments in satisfaction with actions.

On the other hand, depression scores did not significantly improve, in line with findings from other studies on ACT interventions for chronic pain conditions (Chisari et al., 2022). Nor were there any significant changes in outcomes related with pain intensity, either questionnaire-based or ES-based. Most participants evaluated their pain as strong, both before and after the intervention. This result is not unexpected considering the very nature of FM and the characteristics and purpose of the ACT intervention. FM is considered an unresolvable primary chronic pain condition wherein evaluations of affective pain appear to be more susceptible to change after intervention than general evaluations of pain that include permanent sensory symptoms (Clauw, 2015, Wolfe et al., 1990, Wolfe et al., 2010). It is worth noting that participants in this study had been living with pain for the last 20 years or more, despite repeated, prolonged treatment (medical or otherwise) aimed at reducing pain. Besides, the main goal of the ACT intervention was not pain reduction. As the 12th Division of the American Psychological Association (APA) indicates on ACT philosophy and purpose: *“ACT does not seek to cure or control pain or other symptoms as a primary aim…”*, but “… *helping patients to acquire effective behavior patterns guided by what they hold as important”* (McCracken, 2015).

Regarding process measures, results showed significant changes in questionnaire-based measures of pain-related process variables. Specifically, pain acceptance increased and psychological inflexibility in pain decreased, with most participants presenting a clinically significant improvement for these variables. These results are consistent with findings from prior applications of ACT to FM (Ljótsson et al., 2014; J. Luciano et al., 2014; Simister et al., 2018; Wicksell et al., 2013). However, generic (non-specific to pain) process measures did not change significantly after intervention (still, a third of the participants showed clinically significant decreases in experiential avoidance or cognitive fusion). This discrepancy is not uncommon in the ACT literature for specific health conditions, wherein domain-specific PF measures appear to be sensitive to intervention and mediate treatment outcome where a generic PF measure does not (Ong et al., 2019). In any case, it is worth noting that pre-intervention AAQ-II and CFQ scores were not particularly high (below the average for clinical samples), and post-intervention scores were near the average range for general population (Ruiz et al., 2013). In turn, ES-based process measures changed accordingly, with significant reductions in avoidance, inaction, and rumination (both pain-related and emotional-discomfort-related) and a significant increase in valued actions. The most relevant individual changes in PF-related processes were for pain avoidance and discomfort-related inaction, with four and five participants presenting clinically significant pre-post reductions, respectively.

Overall, these findings show that, despite its brevity, the online ACT intervention was useful in moving change-processes in the expected direction, which corresponds with its effects on outcome measures. The intervention promoted the acceptance of pain and emotional discomfort as part of the lives of FM patients, as well as their engagement with valued actions. These changes entailed a reduction of emotional pain and discomfort, as well as of the general impact of FM on the patients' lives. Likewise, it entailed an increase in the patients' satisfaction with undertaken actions. These intervention effects were clearly observed upon group-based analyses, which conveyed a rather homogeneous picture, with apparent improvements (to some degree) in most of the outcomes and processes. Individual analyses, however, revealed a more complex pattern characterized by considerable variability, especially for ES-based measures. These analyses, however, allowed for a more precise identification of those patients for whom the intervention was particularly effective (or otherwise), and of the specific processes of change that were influenced.

Beyond intervention feasibility and preliminary effectiveness, this study aimed to examine the influence of processes of change over momentary outcomes throughout an extended six-week period of assessment after intervention (post-intervention ES). Accordingly, multilevel analyses explored whether, and to which extent, momentary PF-processes predicted momentary clinical outcomes during this period above and beyond questionnaire-based outcome measures immediately after intervention. For each clinical outcome measured through ES, analyses showed that a momentary PF process was sufficient (and superior to any questionnaire-based outcome) in accounting for the variance of the outcome. Pain-related inaction (i.e., the extent to which pain is viewed as a barrier that impedes engagement in valued actions) was sufficient in itself to significantly predict pain intensity in the patients' natural context, without any questionnaire-based outcome measure adding significantly to accounted-for variance. Similarly, discomfort-related inaction (i.e., the extent to which emotional discomfort is viewed as a barrier that impedes engagement in valued actions) sufficed in itself to significantly predict levels of emotional discomfort. Likewise, engagement in valued actions sufficed to significantly predict satisfaction with actions. Therefore, pain (either evaluative or affective), depression, anxiety, and even FM impact (as measured by questionnaires immediately after intervention) failed to add any predictive power to momentarily assessed PF-processes in accounting for the patients' momentary clinical-outcome state in their natural context. Additionally, it has to be noted that the better fit of 1Lv-RC models also entails that the degree of influence of momentary PF-processes on patients' momentary clinical-outcome state would be variable in each participant, though significant in all of them.

These analyses assume an a priori directionality in the relationship between the variables designated as process (those that were targeted for change in intervention) and those designated as outcome (those expected to change upon changes in process). It can be argued that it is difficult to determine the direction of the relationship between a specific process and a specific outcome. For instance, regarding the relationship between pain-related inaction and pain intensity, it was observed that participants who were more blocked in their actions by the experience of pain (i.e., those more inflexibly framing pain as a barrier) also reported more intense pain. It could be argued that the more inflexibly pain is framed (as a barrier that impedes engaging in valued actions while experienced) the more intense it will be perceived to be. Likewise, it could be argued that it is the intensity of pain that actually determines how inflexibly we respond to it (i.e., the more intense the pain, the more likely it will be seen as a barrier for actions). And of course, we can assume a bi-directionality in the relationship between these variables were each of them feeds back on the other. The first interpretation is consistent with the purpose of the ACT intervention (i.e., promoting flexibility regarding pain in order to observe relevant changes in valued action) and seems to be supported by experimental research directly manipulating the way discomfort is framed. For instance, C. Luciano et al. (2010) investigated this in an experimental-analogue study. Participants performed a point-earning task wherein they were intermittently exposed to loud, distressing noises. One group was trained to frame the loud noises as if they were in opposition to performing the task (i.e., as an actual barrier that had to be eliminated before they could go on with the task), while the other was trained to frame the loud noises as in coordination with performing the task (i.e., as something they could experience while they kept performing). Although both groups were equaled in terms of noise exposure (loudness, duration, etc.) and points earned, the first group reported the noises to be significantly more distressing than the second. These findings showed that when discomfort is framed as a barrier (i.e., an analogue of experiential avoidance), it is experienced more intensely than when it is framed as in coordination with valued actions (i.e., an analogue of psychological flexibility). It is also worth considering that the participants in our study had a long history of futile attempts to effect change in the other direction, that is, attempting to reduce pain in order to be able to function (e.g. by taking analgesic medication). Adding further support to the view that change in PF-processes effected change in clinical outcome, ES-based process measures were not only predictive of momentary outcomes during the same temporal period, but also predicted general impact of FM at the 6-month follow-up. Indeed, they were more predictive than FM impact itself at post-treatment.

The use on an idiographic approach with the time-extensive collection of repeated process and outcome measures provided a wealth of information useful in understanding treatment effects linked to processes of change with a small sample, in line with a process-based therapy (PBT) framework (Hayes and Hofmann, 2018; Hofmann and Hayes, 2019). Nonetheless, it is important to mention some limitations of this study that should be addressed in future research in order to enhance the generalizability and scope of these findings. First, experimental control could be improved in future studies with a randomly assigned baseline duration across participants. This form of non-concurrent multiple-baseline single-case design would contribute to attaining a more rigorous control of the extent to which clinically significant change can be directly attributed to treatment. Second, it is possible that the superiority of ES process measures in predicting ES outcome measures (compared to questionnaire-based outcome measures), might be at least partially explained by the fact that both predictor and outcome were collected at the same time and with the same method (i.e., post-intervention ES). However, it should also be noted that both ES and questionnaire-based outcome measures tapped into similar clinical variables; thus, a stronger association amongst the different outcome measures could be expected compared to the association of ES outcome and process measures. Third, it is necessary to take significant effects (or lack thereof) with caution, since the likelihood of Type 1 and 2 errors is high (especially in questionnaire-based outcomes) as a consequence of the small sample size (*n* = 9). Fourth, although our ES-based repeated measures were extended in time, covering a significant period pre- and post-intervention, they were relatively low-density compared to other forms of ES that contemplate several measurements a day. While this might have limited the scope of assessment in terms of sampling less situations, hence providing less access to a wider variety of contexts, it also possibly facilitated higher completion rates than a more demanding ES schedule. Indeed, the completion rate for ES measures was 100 %, which appears to be important considering that FM is characterized by high levels of fatigue, a potential source of participant non-compliance. In addition to the relatively low-demanding ES schedule, the high completion rate can be accounted for by other features of the intervention. All participants were self-referred from a patient-volunteer list (Arndt et al., 2020). The first contact with the therapist and ES completion instructions were individually carried out on the phone, and the therapist provided technical assistance for ensuing potential difficulties with the software tools. Finally, it might as well be that in the context of the COVID-19 social and mobility restrictions standing in Spain at the time, the intervention became a more attractive and reinforcing activity than in other conditions.

## 5 Conclusions

In sum, the present study presented a brief, online group ACT intervention for FM patients that was feasible and preliminarily effective in producing clinical benefits, both in terms of formally measured, questionnaire-based variables, and of momentary, in-context outcome and process measures. Smartphone-based ES allowed for a more ecological data collection, showing that post-treatment reductions in emotional discomfort and increments in satisfaction with actions were maintained for most of the participants in their natural contexts along the 6-week post-intervention period, and that these effects were mainly influenced by PF-related processes in the same contexts. In addition, the latter successfully predicted the longer term impact of FM at follow-up. We believe that an idiographic approach to the study of ACT-based interventions for chronic pain will contribute to a better understanding of the processes of change that can be effectively targeted in ever more personalized interventions.

## CRediT authorship contribution statement

All authors contributed to the study conception. Clinical intervention was designed by P.d.l.C., M.R.V., and M.H.L., conducted by P.d.l.C. and supervised by M.R.V., and M.H.L. Material preparation, data collection, analysis and the first draft of manuscript was written by P.d.l.C. Comments and edits on the manuscript were performed by M.R.V., and M.H.L. All authors read and approved the final manuscript.

## Ethical standards

This study was approved by the Ethics Committee of the University of Jaén according to the declaration of Helsinki of 1964, as revised in 2013.

## Funding

This clinical research work has been supported by a postdoctoral fellow from Andalusian government co-financed by 10.13039/501100004895European Social Funds (Ref: DOC2020-00462).

## Declaration of competing interest

All authors declare that they have no conflict of interest.
